# Evaluation of serum miR-122-5p, miR-486-5p, miR-21-5p, and their interplay with inflammatory markers in diabetic nephropathy: a case control study

**DOI:** 10.1186/s12882-025-04619-w

**Published:** 2025-11-29

**Authors:** Enas Ahmed Osman, Noha Hamdy Eltaweel, Medhat Madbouly, Osama Mosbah, Mohammed Ghanem Gaber, Farida Mohamed Khanany

**Affiliations:** 1 Clinical Chemistry Department, Theodor Bilharz Research Institute, Giza, Egypt; 2 Medical Molecular Genetics Department, National Research Centre, Cairo, Egypt; 3 Radiology Department, Theodor Bilharz Research Institute, Giza, Egypt; 4 Nephrology Department, Theodor Bilharz Research Institute, Cairo, Egypt

**Keywords:** DKD, miRNA-122-5p, miRNA-21-5p, miRNA-486-5p, Biomarkers, Diagnosis

## Abstract

The development of diabetic kidney disease (DKD), a major consequence of type 2 diabetes mellitus (T2DM), is impacted by hereditary factors, immunological dysregulation, and inflammation. Inflammatory indicators and circulating microRNAs (miRNAs) can be stable and used as diagnostic biomarkers. The objective of this study was to assess whether serum miRNA-122-5p, miRNA-486-5p, and miRNA-21-5p can serve as non-invasive diagnostic biomarkers for DKD by comparing their levels in T2DM patients with and without DKD against healthy controls, and by examining their correlations with inflammatory markers (IL-6, TNF-α, and hsCRP). Finally, their role in DKD pathogenesis was also investigated through their possible target genes using a bioinformatic analysis. A total of 1350 individuals were enrolled: 450 T2DM with DKD, 450 with T2DM without DKD, and 450 healthy controls. We used qRT-PCR to quantify miRNA expression, ELISA to assess IL-6 and TNF-α, and an immunoturbidimetric technique to detect hsCRP. We found that miRNA-122-5p and miRNA-21-5p levels were substantially elevated in DKD patients and positively correlated with hsCRP and TNF-α; miRNA-21-5p also correlated with IL-6. Conversely, miRNA-486-5p expression was markedly reduced in the DKD group and showed no correlation with inflammatory markers. Enrichment analysis revealed involvement in TNF signaling, insulin resistance, and growth hormone pathways. Their differential expression in DKD may support the utility of miRNA-122-5p, miRNA-21-5p, and miRNA-486-5p expression as potential biomarkers for DKD.

## Introduction

Type 2 diabetes mellitus (T2DM) is among the most common metabolic disorders globally, with an increasing frequency [1]. A major microvascular consequence of T2DM, diabetic kidney disease (DKD), contributes considerably to morbidity and is linked to a 31.1% rise in mortality [2]. The International Diabetes Federation reports that diabetes is a major contributor to chronic kidney disease (CKD) and that, when coupled with hypertension, accounts for almost 80% of all occurrences of end-stage renal disease worldwide [3, 4].

Expanding and hypertrophying glomerular mesangial capillaries, the buildup of extracellular matrix proteins, and worsening podocyte dysfunction are hallmarks of DKD [2]. The development of advanced glycation end products and the combined effects of chronic hyperglycemia, inflammation, and oxidative stress drive these pathological alterations by encouraging the recruitment and activation of immune cells inside the renal interstitium [5].

The infiltrating immune cells, primarily macrophages and lymphocytes, release increased amounts of pro-inflammatory cytokines, like interleukin-6 (IL-6), and growth factors, such as tumor necrosis factor-alpha (TNF-α). These substances exacerbate tissue inflammation and damage [6]. In response to this inflammatory cascade, the liver synthesizes and releases C-reactive protein (CRP) [7].

All these variables trigger renal tubule apoptosis and fibrosis, diminishing kidney function. Considering the proven significance of inflammation in diabetic Kidney disease (DKD) progression, understanding the molecular mechanisms that facilitate this inflammatory response is crucial for developing targeted therapeutics [8].

Non-coding RNA molecules around 22 nucleotides in length are called microRNAs (miRNAs) [9]. Their important roles in apoptosis, cell proliferation, immunological responses, and other biological processes have led to their recognition as post-transcriptional regulators of gene expression in recent years [10]. Nearly one-third of all genes that code for proteins are regulated by microRNAs (miRNAs), which are encoded by an estimated 5% of the human genome [11].

In DKD, the miRNA expression profile undergoes significant alterations, suggesting a potential role as biomarkers of DKD. However, the exact molecular processes that cause these alterations are still not well known [11].

In humans, the miRNA known as MiR-21 is located on chromosome 17 at 17q23.1. Its promoter region contains multiple enhancer elements with binding sites for various regulatory targets [12]. The regulatory intricacy of miR-21 has piqued the curiosity of academics seeking to understand its biological significance in relation to DKD [12].

Similarly, serum miRNA-486 is among the primary miRNAs identified as having an active regulatory role, with aberrant expression in several clinical situations [13]. MiR-486 is encoded by the miR486-1 gene, which is located on chromosome 8 at the 8p11.21 region of the human genome [14]. MiRNA-486 has been shown to influence the production of inflammatory cytokines, which are known to play a role in the progression of diabetic neuropathy [14].

In humans, miR-122 is mostly expressed in the liver and is encoded at a single genomic location on chromosome 18 [15]. Research has shown that miR-122 is markedly elevated in renal tubular cells in mice that had artificially induced DKD in their kidneys [16]. Consistent with these findings, previous studies in adults have reported similar expression patterns for miR-122-5p, as well as for miR-486-5p and miR-21-5p, highlighting their potential relevance in kidney pathology [11, 12, 15].

To determine if these miRNAs are useful diagnostic biomarkers for DKD, this study compared their serum levels in T2DM patients with and without DKD vs. those of healthy persons. The study also examined any associations between these miRNAs and certain inflammatory indicators. An in-silico study was carried out to find important target genes mediating the effects of the examined miRNAs and investigate their role in the pathogenesis of DKD. This would help us better grasp their biological relevance.

## Methods

### Ethical approval

The study protocol was approved by the Institutional Review Board (IRB) of Theodor Bilharz Research Institute (FWA00010609) before it was started, and it was recorded under the serial number PT (687). All participants were required to provide written informed consent following IRB requirements. The study adhered to the ethical standards in the Declaration of Helsinki and its updates.

### Study design and participants

Between November 2022 and May 2025, 1,350 Egyptians were enrolled in this case-control study at the Theodor Bilharz Research Institute’s Nephrology Department and Outpatient Clinic. Each subject was randomly assigned to one of three groups (*n* = 450 each): Group (A) patients with DKD at stages II, III, and IV, Group (B) patients with T2DM without DKD; and Group (C) healthy controls, matched racially, with no personal or family history of T2DM selected randomly from blood donors. A thorough clinical assessment was conducted on all patients, which included taking their medical history, doing a physical examination, and using imaging techniques such as renal Doppler and ultrasonography.

#### Inclusion criteria

The following criteria were used to obtain a T2DM diagnosis, following the American Diabetes Association (ADA) [17]: Hyperglycemia or a crisis may be indicated by a random plasma glucose level of 200 mg/dL, a fasting plasma glucose level of 126 mg/dL, or a HbA1c level of 6.5% or above.

Dialysis-related complications were categorized using the Kidney Disease Improving Global Outcomes (KDIGO) criteria for stages II-IV of CKD [18]. Diabetic kidney disease was defined as either a continuous decrease in eGFR or a continuous increase in urine albumin-to-creatinine ratio (>30 mg/g) on two separate occasions, spaced at least three months apart.

#### Exclusion criteria

Exclusion criteria included a history of kidney transplantation, autoimmune disorders, refractory hypertension, cardiovascular illness, liver disease, hemodialysis, immunosuppressive medication, cancer, or CKD caused by a non-diabetic factor.

### Laboratory investigations

All blood samples were collected from subjects in the morning following a 10–12 h overnight fast as part of their routine clinical follow-up. For Routine Biochemistry and Inflammatory Markers: Four milliliters of venous blood were collected into plain sterile vacutainers. These samples were allowed to clot at room temperature, centrifuged at 4500 rpm for 5 min to obtain serum. The serum was then aliquoted: One aliquot was used immediately for routine analysis (urea, creatinine, uric acid, albumin, fasting blood glucose (FBS). Remaining aliquots for hsCRP, IL-6, and TNF-α were immediately stored at − 80 °C until analysis to maintain cytokine integrity and minimize degradation. For HbA1c, two milliliters of venous blood were collected separately in EDTA vacutainers for the determination of HbA1c. Urine Samples: Spot urine samples were also obtained to assess the albumin-to-creatinine ratio (ACR), patients were provided with specific instructions on proper collection techniques.

The estimated glomerular filtration rate (eGFR) was calculated by integrating laboratory measurements with patient demographic data, using the 2021 CKD-EPI equation (Chronic Kidney Disease Epidemiology Collaboration) [19].

### Measurement of serum IL-6 and TNF-α

The quantities of IL-6 and TNF-α in the serum were measured using ELISA kits from BT LAB, China (IL-6 Cat. No. E0090Hu, TNF-α Cat. No. E0082Hu), according to the instructions provided by the manufacturer. Reducing the number of freeze-thaw cycles throughout the processing of the samples was a primary goal.

### Gene expression analysis

#### Serum preparation and miRNA extraction

To preserve RNA integrity, blood samples for miRNA analysis were collected in separate tubes either than samples obtained for biochemical tests. Samples were processed within one hour of collection to minimize RNA degradation. Following centrifugation at 4500 rpm for 10 min, the obtained serum was promptly aliquoted into sterile cryovials and stored at − 80 °C until RNA extraction. The miRNeasy Serum/Plasma Kit (Qiagen) was used to separate total RNA, including miRNAs, from serum. The resulting sample was then kept at -70 °C. Each sample was supplemented with 2 µL of a synthetic C. elegans miR-39 miRNA mimic to evaluate the extraction and reverse transcription effectiveness. The concentration and purity of the RNA were assessed at 260/280 nm using a NanoDrop spectrophotometer.

#### qRT-PCR analysis

The Advanced miRNA cDNA Synthesis Kit (TaqMan, Thermo Scientific) was utilized for reverse transcription after polyadenylation tailing was applied to 10 ng of isolated RNA. To synthesize cDNA for miRNA-21-5p, miRNA-122-5p, and miRNA-486-5p, we utilized a universal RT primer included in the kit.

The QuantStudioTM 5 Real-Time PCR System was utilized to measure expression levels. Relative quantification was measured using a TaqMan master mix, gene-specific TaqMan probes, the C. elegans miR-39 mimic was used as a spike-in control. The relative expression (Rq) was determined using the ∆∆CT technique, after normalizing the cycle threshold (CT) values against reference genes [20].

### Bioinformatic analyses

#### Target gene prediction

The miRWalk v.3 program (http://mirwalk.umm.uni-heidelberg.de/) was used to forecast which genes are possible targets of the studied miRNAs. Genes selected were those found in at least one target prediction tool (TargetScan or miRTarBase). To assess their relevance to DKD, DKD-related genes were extracted from DisGeNET via Enrichr (https://maayanlab.cloud/Enrichr/#) and compared with the predicted targets of the studied miRNAs.

#### Functional enrichment analysis

The list of target genes was functionally annotated using Enrichr. A pathway analysis was carried out using the KEGG database to determine the highly enriched pathways affected by the list of target genes.

#### Protein-protein interaction and network construction

We examined the protein-protein interactions (PPIs) among those target genes associated with DKD, using the STRING database. Confidence ratings were used to classify interactions as either “highest” (> 0.9), “high” (0.7–0.9), or “medium” (0.4–0.7). Interactions scoring < 0.4 were excluded. The resulting network was visualized using Cytoscape.

### Statistical analysis

IBM SPSS Statistics version 27.0 was employed to process and analyze the data. Mean ± SD was used to describe parametric data, whilst median and IQR were used to portray non-parametric data. When comparing groups, categorical data were tested using chi-square tests, parametric continuous variables with independent t-tests, and non-parametric data with the Kruskal-Wallis test. We applied Spearman’s correlation to correlate between the continuous variables. We calculated the best cutoff values using receiver operating characteristic (ROC) curve analysis and provided the results along with specificity, sensitivity, and area under the curve (AUC). With a 95% confidence interval, a p-value less than 0.05 was deemed to indicate statistical significance.

## Results

### Study groups’ demographics and laboratory results

This study included a total of 1,350 participants, categorized into three groups: Group (A) consisted of 450 patients with DKD at stages II, III, and IV; Group (B) included 450 patients with T2DM without DKD; and Group (C) comprised 450 healthy, racially matched controls with no personal or family history of T2DM, randomly selected from blood donors. When comparing the groups according to gender (p-value = 0.920) or age (p-value = 0.069), no statistical significant differences were found.

kidney function was largely preserved in controls and T2DM patients without DKD. In contrast, DKD patients showed lower serum albumin, higher urea and creatinine, albumin/creatinine ratio and markedly lower eGFR compared with the other groups. FBG and HbA1c were significantly higher in both diabetic groups compared with controls, as shown in Table 1. Furthermore, when contrasted with the T2DM and controls, the DKD group showed noticeably higher levels of hsCRP, IL-6, and TNF-α, as shown in Table 2.

### Comparison of miRNA expression in the research groups

The DKD group had substantially higher serum miRNA-122 expression levels compared to the T2DM group and healthy controls, moreover the T2DM group also showed a significant increase compared to controls. Similarly, there was a substantial increase in serum miRNA-21-5p expression in the T2DM group compared to controls and the DKD group, the T2DM group also showed considerably higher levels than controls. On the other hand, serum miRNA-486-5p expression was considerably lower in the DKD group compared with both the controls and T2DM group, the T2DM group also showed significant decreased levels compared to the controls as shown in Table 3.

### Correlation between individual miRNAs and inflammatory parameters in the DKD group

**Fig. 1:**
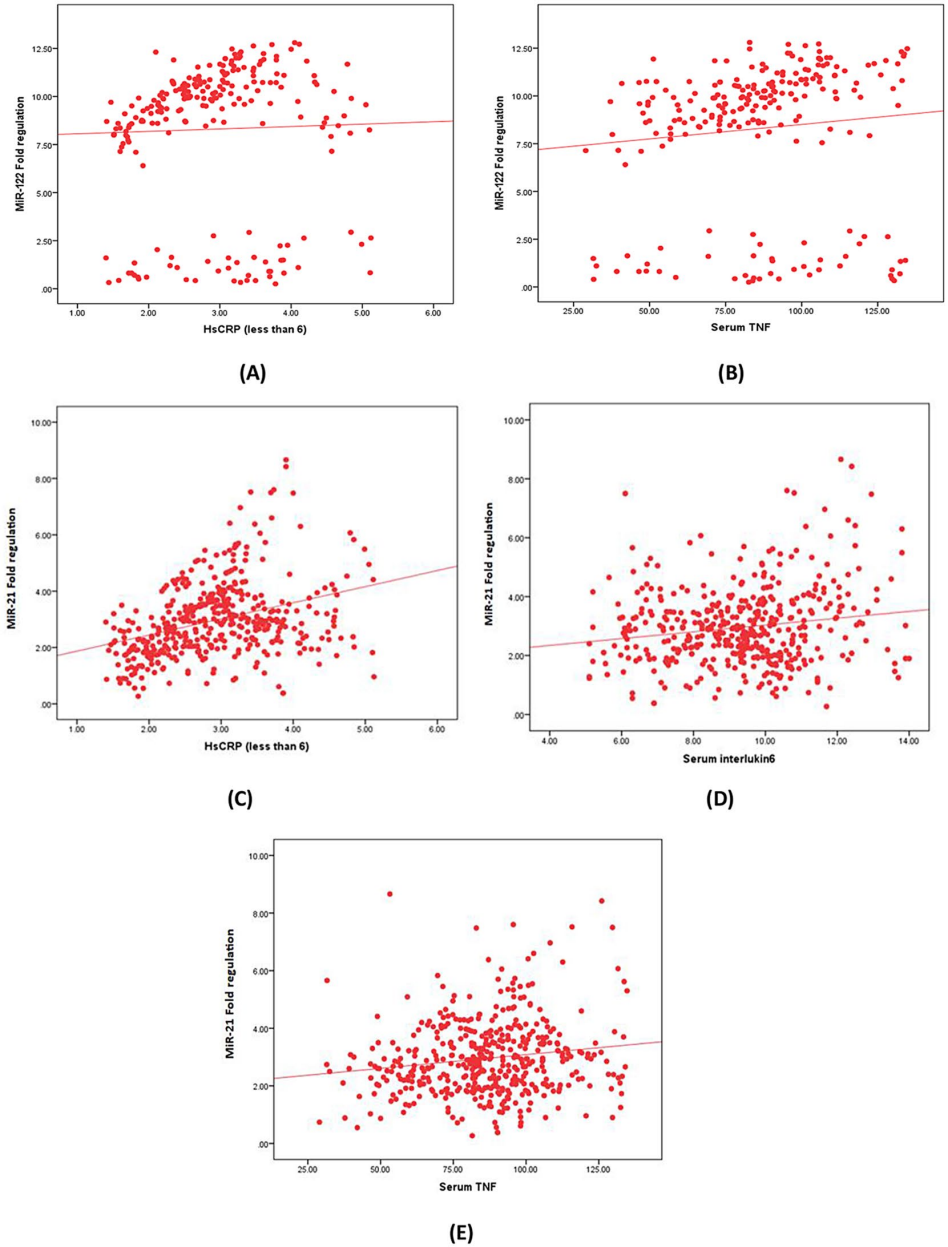
Correlation between the studied miRNAs and inflammatory parameters among the DKD group. (**A**) Correlation between serum miR-122-5p and HsCRP, (**B**) Correlation between serum miR-122-5p and TNF-α, (**C**) Correlation between serum miR-21-5p and HsCRP, (**D**) Correlation between serum miR-21-5p and IL-6, (**E**) Correlation between serum miR-21-5p and TNF-α among the diabetic kidney disease group

Serum hsCRP and TNF-α were positively correlated with miRNA-122-5p, also hsCRP, IL-6, and TNF-α were positively correlated with miRNA-21-5p. On the other hand, there was no significant correlation between miRNA-486-5p and the tested inflammatory markers, as shown in Table 4; Fig. 1**.**

### Diagnostic value of serum MiRNAs in DKD

#### ROC curve analysis

**Fig. 2:**
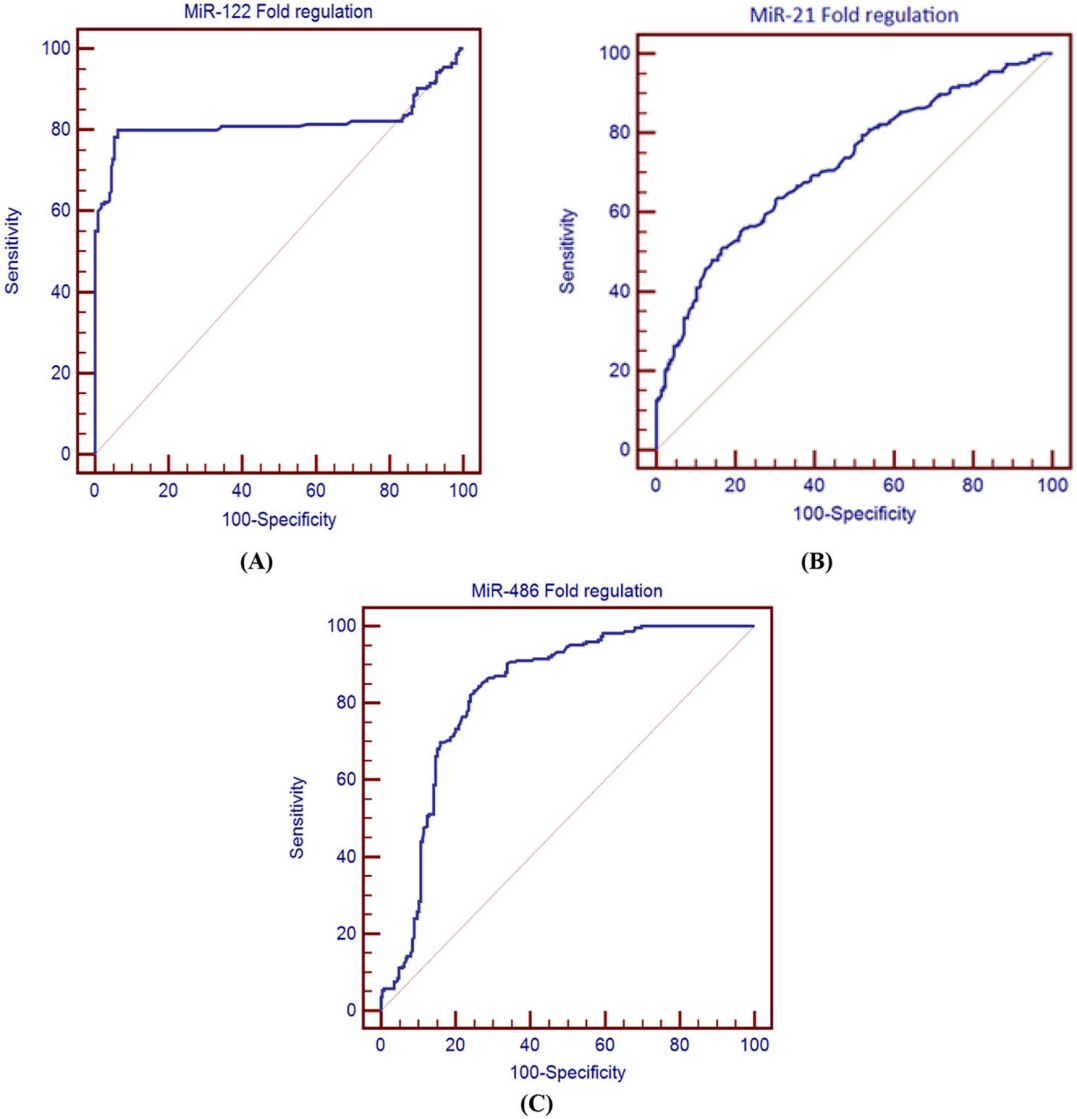
ROC curve analysis (**A**) miR-122-5p, (**B**) miR-486-5p, (**C**) miR-21-5p to differentiate between patients with T2DM with and without DKD

Receiver operating characteristic curve analysis demonstrated that all three studied miRNAs showed good diagnostic performance in distinguishing T2DM with DKD, from those without. Comparing to miR-21-5p and miR-486-5p, the results of the ROC curve showed that serum miR-122-5p had the best diagnostic accuracy with an AUC of (0.861 vs. 0.827 and 0.774, respectively), as shown in Table 5, Fig. 2**.**

### Bioinformatic analyses

#### Target gene prediction

According to DisGeNet, 560 genes are associated with DKD. Among these, 22 genes were identified as putative targets of at least one of the studied miRNAs. The number of target genes associated with DKD’s pathogenesis predicted as targets for miR-122-5p, miR-21-5p, and miR-486-5p were 5, 12, and 5 genes respectively, Table 6.

#### Analysis of functional enrichment

**Fig. 3:**
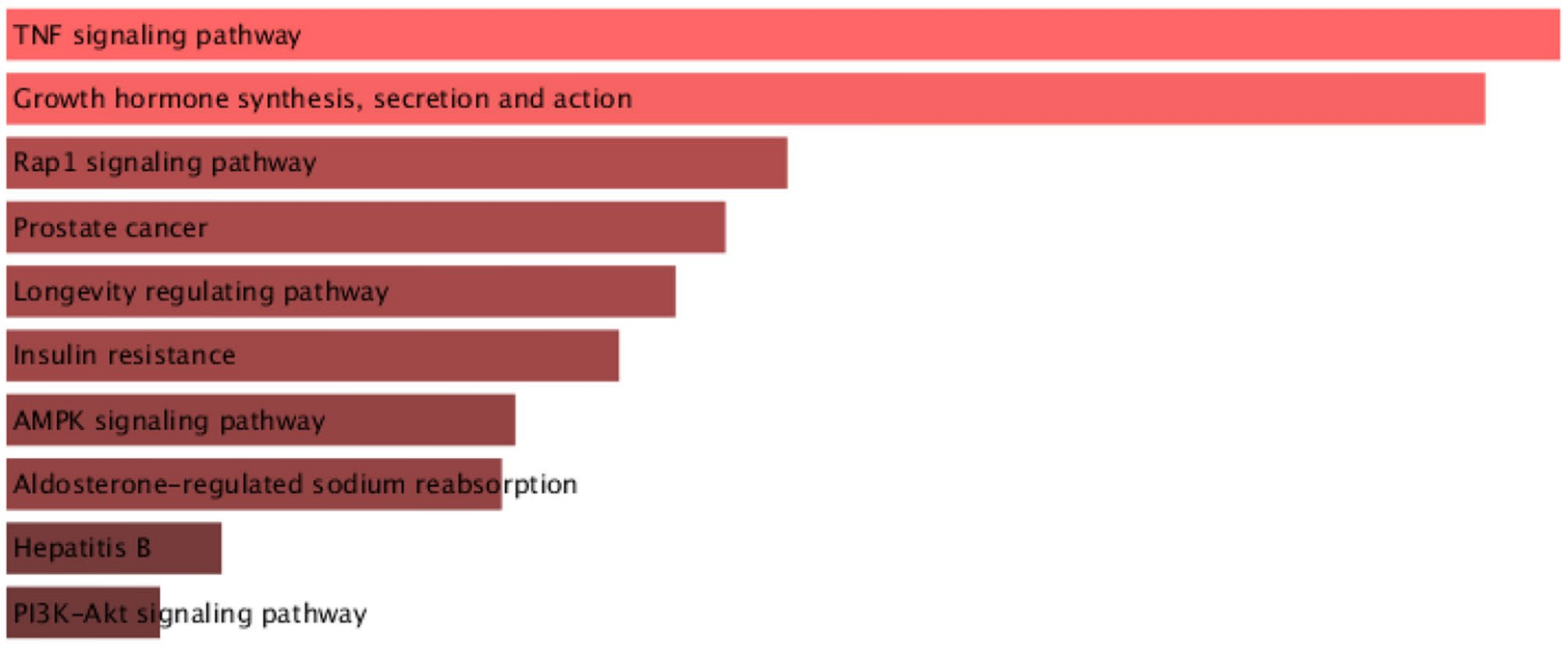
Enriched pathways affected by the DKD gene list, sorted according to their p-value. Colors primarily indicate the significance of enriched terms and the source of the gene set library. Longer and lighter bars generally represent higher significance

Using Enrichr and the KEGG database, we performed functional enrichment analysis on the list of target genes. The DKD-related genes were found to be involved in several enriched pathways, including TNF signaling, growth hormone (GH) synthesis and action, insulin resistance (IR), and other signaling cascades. The pathways are ranked based on their p-values in Fig. 3.

#### Protein-protein interaction (PPIs) and network construction

**Fig. 4:**
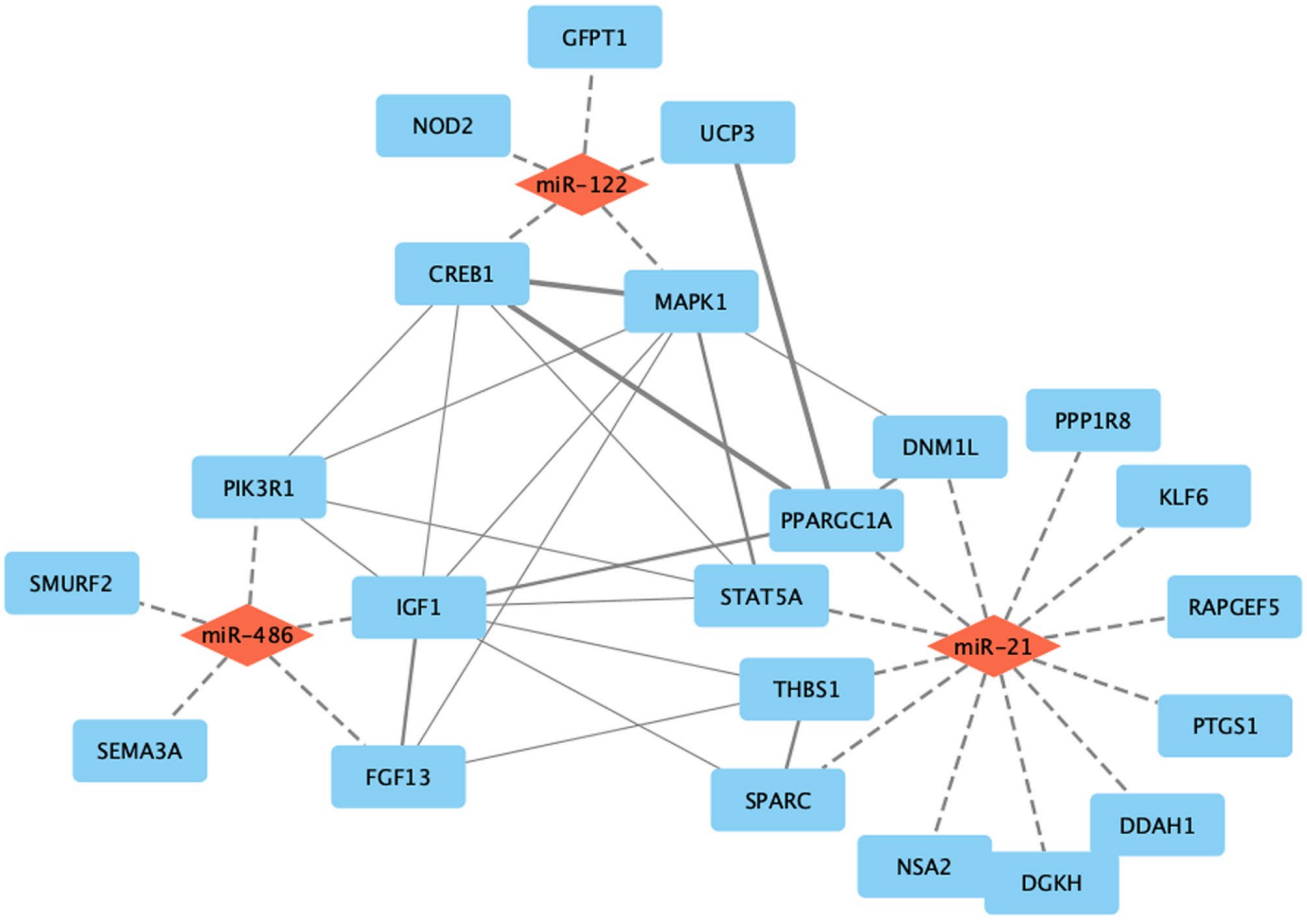
Network showing the studied miRNAs with their target genes and possible PPI between them. miRNAs are in red while genes are in blue. Dashed edges are predicted miRNA-genes targeting relationship, while solid lines are PPI with different widths showing variable confidence

Potential interactions among the selected target genes (related to DKD) were investigated by analyzing PPIs using STRING database, as shown in Table 7, and Fig. 4. Only interactions with a score greater than 0.4 were selected. The selected interactions were constructed and visualized using Cytoscape.

## Discussion

Inflammation is a key driver of DKD onset and progression, with pro-inflammatory cytokines and markers promoting renal damage and fibrosis [4]. Altered expressions of (miR-122-5p, miR-486-5p, and miR-21-5p) in diabetic nephropathy suggest their involvement in these processes, while the selected inflammatory markers (IL-6, TNF-α, CRP) mediate DKD-related inflammation. Investigating their relationship may clarify key mechanisms driving DKD and highlight their potential as diagnostic biomarkers [6].

In this study, serum miR-122-5p level was significantly higher in DKD patients than in T2DM patients and healthy controls, supporting its role as a potential biomarker of diabetic kidney disease. This agrees with Regmi et al. [2] and Zeinali et al. [21], who reported elevated miR-122-5p in DKD and T2DM, respectively. The observed upregulation may contribute to DKD progression by suppressing AMP-activated protein kinase (AMPK) activity [22], promoting lipid and glycogen accumulation in renal tissue [23]. Interestingly, animal models have shown a protective effect of miR-122-5p overexpression, indicating that its role may differ across species [23]. The positive correlations between miR-122-5p and hsCRP and TNF-α in our study further suggest a link with inflammatory activation [21]. This might be explained by research showing that upregulation of miR-122-5p triggers phosphorylation and activation of protein kinase B, increasing the cytokine production [24].

Based on our findings, serum miRNA-21-5p expression was considerably greater in the DKD group compared to the T2DM group and normal controls, and in the T2DM group compared to normal controls. In line with this finding, recent studies found that patients with DKD and overt proteinuria had higher levels of miRNA-21-5p than those with T2DM and normo-albuminuria [25]. The profibrotic effect of miR-21-5p may be attributed to its suppression of key regulatory genes, such as phosphatase and TENsin homolog (PTEN) and SMAD family member 7 (SMAD7) [26]. Animal models of DKD have demonstrated protective advantages of inhibiting miR-21-5p, which reduces microalbuminuria, renal fibrosis, and inflammation [27].

We found that miRNA-21-5p was positively correlated with serum hsCRP, IL-6, and TNF-α, which means that miR-21-5p could be involved in the inflammatory processes that lead to DKD. Consistent with a recent research conducted in Turkey [28] performed on a group of individuals undergoing hemodialysis, the results showed a strong positive association between miRNA-21-5p and serum IL-6 and TNF-α.

Our current study found that serum miR-486-5p expression was lower in the T2DM group than in normal controls and the DKD group; furthermore, the T2DM group had significantly lower serum miR-486-5p expression than controls. Regmi et al. [2] found that serum miR-486-5p expression was significantly lower in the DKD group compared to the T2DM group and healthy controls, which aligns with our findings. Researchers suggested that miR-486-5p’s anti-fibrotic effects in DKD were due to its targeting of NFAT5 [29], a protein that plays a pathogenic role in the disease by increasing the activity of nuclear factor (NF)-κB, which in turn promotes the expression of genes related to angiogenesis, platelet activation, vascular smooth muscle cell and macrophage migration, and pro-inflammatory cytokine expression [30].

A bioinformatic analysis was conducted to determine the importance of the miRNAs under study in the development of DKD. There are 22 putative target genes for the investigated miRNAs, that are implicated in DKD. In Table 5, it was noted that 12 genes are anticipated to be targeted by miR-21-5p, five genes by miR-122-5p, and five more genes by miR-486-5p. Those genes might be crucial affecting the role of those studied miRNAs in the pathogenesis of DKD.

MiR-21-5p was expected to target 12 key genes involved in DKD, e.g., DGKH, DDAH1, DNM1L, PPARGC1A, STAT5, THBS1, and SPARC [31]. Among the renoprotective genes, miR-21-5p is predicted to target dimethylarginine dimethylaminohydrolase 1 (DDAH1), Dynamin 1-like protein (DNM1L) peroxisome proliferator-activated receptor gamma coactivator 1 alpha (PPARGC1A), and diacylglycerol kinase eta (DGKH), whose gene expression was previously demonstrated to be markedly reduced in diabetic nephropathic rats [32, 33]. Also, DDAH1 is essential for maintaining renal function, especially in diabetes and chronic kidney disease, and increasing its expression in kidney can alleviate DKD symptoms [34].

In the same context, being protective against renal injury in DKD, the stability of mitochondria is essentially controlled by two genes, DNM1L and PPARGC1A, which are also known as PGC1-α. Suppression of DNM1L in DKD can decrease tubular cell death and alleviate kidney damage, suggesting that it may have therapeutic relevance [35, 36]. On the other hand, the ligand-activated nuclear hormone receptor superfamily gene PPARGs is known to protect against DKD and other kidney disorders [37]. PPARGC1A is significantly downregulated within the kidney in DKD, contributing to diminished mitochondrial biogenesis and increased mitochondrial oxidative stress [38]. Sustained PPARGC1A expression preserves mitochondrial function and confer renoprotective effects in animal models of DKD [39]. Therefore, the elevated miR-21-5p might suppress those renoprotective genes and thus might be a part of the DKD pathogenesis.

Furthermore, three highly expressed proteins implicated in development of diabetic complications and DKD pathogenesis—STAT5A, Thrombospondin-1 (THBS1), and secreted protein acidic and rich in cysteine (SPARC)—are possibly targeted by miR-21-5p. According to previous reports, hyperglycemic cultured cells and DKD rats showed increased STAT5A levels, while silencing STAT5A in-vitro reversed the impact of elevated hyperglycemia [40, 41]. Similarly, THBS1 and SPARC activate an important component in the development of DKD, which is transforming growth factor-β1 (TGF-β1) [42].

From data considered, we could conclude that miR-21-5p regulates a number of critical genes that may affect DKD progression. Thus, it can enhance our knowledge of the pathogenesis of DKD and might be a target for future treatments.

Similarly, many key genes in DKD are predicted targets of miR-486-5p, including insulin-like growth factor-1 (IGF1), phosphoinositide-3-kinase regulatory subunit-1 (PIK3R1), and fibroblast growth factor-13 (FGF13). Normal glucose homeostasis depends on IGF1, such that, individuals with T1DM, T2DM, and DKD often have lower IGF1 levels compared to healthy controls, with somewhat greater levels seen in T2DM than DKD [43]. Therapeutically, metabolic and vascular complications in DKD could be potentially lessened by regulating IGF1 expression [38], which is a putative target of miR-486-5p. In addition, as T2D progresses, glomerular endothelial cells (GECs) exhibit an increase in FGF13 expression. Overexpression of FGF13 worsens T2D-associated nephrotic damage. FGF13’s absence improves mitochondrial homeostasis and maintains endothelial barrier integrity [44]. Another powerful therapeutic target in the development of kidney damage is the signal transducer and activator of transcription 3 (STAT3)/PIK3R1/mechanistic target of rapamycin (mTOR) axis. It is probable that the effects of pharmacologically blocking STAT3 or mTOR, which are targets of miR-486-5p, significantly improves renal function and suppresses inflammation and apoptotic signaling [45]. Thus, miR-486-5p is an intriguing molecular target for the treatment and prevention of DKD.

In the same context, one of the potential targets of miR-122-5p was the cAMP response element binding protein 1 (CREB1), and mitogen-activated protein kinase 1 (MAPK1) which are crucial in the pathological sequence of DKD. Several studies demonstrated that CREB1 is activated in early-stage DKD, suggesting that CREB1 activation may be involved in early kidney injury [42]. Overexpression of miR-122 acts by directly targeting CREB1 in gastric and liver cancers [46, 47]. In addition, CREB1 targeting via miR-10a overexpression reduced kidney injury [48]. In the same context, MAPK1’s expression is markedly upregulated under hyperglycemic conditions [49]. Modulating the MAPK/ERK signaling pathway through specific miRNAs—such as miR-133—holds therapeutic promise in attenuating kidney injury associated with DKD [50]. Interestingly, CREB1 has been recognized as a downstream effector within the MAPK signaling cascade [51]. Collectively, miRNA-induced inhibition of CREB1 and MAPK, which are potential targets of miR-122-5p, could ameliorate kidney damage in DKD.

Functional annotation of DKD-related genes revealed enrichment in TNF signaling, GH/IGF-1, insulin resistance, and other pathways as shown in Fig. 3. Elevated serum TNF-α in DKD and T2DM supports its role in driving the transition from diabetes to DKD [52]. The GH/IGF-1 axis also influences renal hemodynamics and fibrosis, while insulin resistance accelerates both metabolic and renal deterioration [53, 54]. All these pathways were previously discussed to be crucial in the DKD pathogenesis. These in-silico findings showed that the studied miRNAs participate in key pathogenic pathways of DKD and may point to novel therapeutic targets.

### Limitations of this study

The primary limitations of this study were the recruitment of participants exclusively from a single hospital and a single ethnic group which may limit the generalizability of the findings. The results of the in-silico analysis, including the predicted target genes and enriched pathways, need to be validated through experimental models and longitudinal clinical studies.

## Conclusion

The possibility of miRNA-122-5p and miRNA-21-5p as non-invasive biomarkers for DKD diagnosis is supported by the fact that they are upregulated in DKD patients, whereas miRNA-486-5p is downregulated. Particularly, miR-21-5p correlated with hsCRP, while miR-122-5p correlated with hsCRP and TNF-α, suggesting that they might be involved in the inflammatory cascades that propel the development of DKD. With an emphasis on the functions of the miRNAs under investigation, this in-depth study delves into new information on the molecular pathophysiology of DKD. It outlines the potential protective or pathogenic mechanistic contributions within important kidney cell types, such as podocytes, glomerular endothelial cells, and tubular epithelial cells. By uncovering these intricate molecular interactions, the study offers a foundation for developing novel, miRNA-targeted therapeutic strategies to halt or reverse renal damage in DKD.

**Table 1:** Comparison of laboratory parameters between the DKD group and the T2DM group without DKD

	Control group (*n* = 450)	DKD (*n* = 450)	T2DM without DKD (*n* = 450)	*P*
History of the known duration of diabetes (years)	--	13.91 ± 1.97	9.31 ± 1.34	0.001*
Urea (mg/dl)	21.89 ± 10.56^a^	60.1 ± 19.25^b^	25.95 ± 10.3^c^	0.001*
Creatinine (mg/dl)	0.70 ± 0.09^a^	1.98 ± 0.72^b^	0.72 ± 0.08^a^	0.001*
Uric acid (mg/dl)	4.97 ± 1.00^a^	6.74 ± 1.37^b^	5.14 ± 0.78^c^	0.001*
eGFR	102.75 ± 10.64^a^	40.65 ± 18.61^b^	102.1 ± 8.03^a^	0.001*
Albumin (g/dl)	3.95 ± 0.42^a^	3.12 ± 0.26^b^	3.75 ± 0.29^c^	0.001*
FBG (mg/dl)	82.75 ± 8.30^a^	199.59 ± 38.81^b^	182.48 ± 44.5^c^	0.001*
HBA1c (%)	4.95 ± 0.35^a^	8.91 ± 1.18^b^	8.07 ± 0.98^c^	0.001*
Albumin/creatinine ratio	9.98 ± 6.16^a^	496.72 ± 351.77^b^	12.28 ± 4.68^a^	0.001*

Data are presented as mean ± SD. * Significant P value < 0.05, T2DM: type 2 diabetes mellitus, DKD: diabetic kidney disease, eGFR: estimated glomerular filtration rate, FBG: fasting blood glucose, HbA1c: Hemoglobin A1c, hsCRP: high-sensitivity C-reactive protein. Different small superscript letters indicate significant differences between groups

**Table 2:** Comparison of inflammatory markers among the study groups

	Group (A) DKD (*n* = 450)	Group (B) T2DM without DKD (*n* = 450)	Group (C) Normal Control(*n* = 450)	*P*
HsCRP	2.9 ± 0.79^a^	0.87 ± 0.25^b^	0.56 ± 0.13^c^	0.001*
Serum IL-6 (ng/L)	9.32 ± 1.86^a^	5.04 ± 0.9^b^	1.56 ± 0.42^c^	0.001*
Serum TNF (ng/L)	86.61 ± 20.75^a^	38.83 ± 12.76^b^	14.01 ± 4.5^c^	0.001*

Data are presented as mean ± SD. * Significant P value < 0.05, T2DM: type 2 diabetes mellitus, DKD: Diabetic kidney disease, hsCRP: high-sensitivity C-reactive protein, IL-6: interleukin 6, TNF-α: tumor necrosis factor alpha

Different small superscript letters indicate significant differences between groups

**Table 3:** Relative expression of miR-122-5p, miR-486-5p, and miRNA-21-5p in the studied groups

	Group (A) DKD (*n* = 450)	Group (B) T2DM without DKD (*n* = 450)	Group (C) Normal Control(*n* = 450)	*P*
MiR-122-5p RQ	9.7 (7.99–10.73)^a^	2.8 (2.54–3.03)^b^	0.96 (0.8–1.15)^c^	0.001*
MiR-486-5p RQ	0.37 (0.24–0.49)^a^	0.87 (0.69–1.23)^b^	0.99 (0.43–2.09)^c^	0.001*
MiR-21-5p RQ	2.82 (2.06–3.6)^a^	1.8 (1.28–2.3)^b^	0.47 (0.22–0.8)^c^	0.001*

Data are presented as median (IQR). * Significant P value < 0.05. T2DM: type 2 diabetes mellitus, DKD: Diabetic kidney disease

Different small superscript letters indicate significant differences between groups

**Table 4:** Correlation between serum miR-122-5p, miR-486-5p, and miRNA-21-5p levels and the studied inflammatory markers among the diabetic nephropathy group

	MiR-122-5p Rq		MiR-486-5p Rq		MiR-21-5p Rq	
	*r*	*p*-value	*r*	*p*-value	*r*	*p*-value
HsCRP	0.234**	**0.001**	-0.083	0.079	0.380**	**0.001**
Serum IL-6	-0.069	0.304	0.054	0.252	0.108*	0.021
Serum TNF	0.282**	**0.001**	-0.006	0.903	0.133**	**0.005**

r: Spearman correlation coefficient., * Significant P value < 0.05, hsCRP: high-sensitivity C-reactive protein, IL-6: interleukin 6, TNF-α: tumor necrosis factor alpha

**Table 5:** Receiver operating characteristic analysis of the studied MiRNAs for differentiating T2DM patients with and without diabetic kidney disease

Variable	Cut point	AUC	Sensitivity (%)	Specificity (%)
MiR-122-5p Rq	> 6.95	0.861	76.00	93.98
MiR-486-5p Rq	≤ 0.73	0.774	88.89	63.74
MiR-21-5p Rq	> 1.7	0.827	80.0	71.36

AUC: Area under the curve

**Table 6:** Genes involved in DKD that are putative target genes of one of the studied MiRNAs

	Gene Symbol	miRNA ID
Diabetic nephropathy	CREB1	hsa-miR-122-5p
	DDAH1	hsa-miR-21-5p
	DGKH	hsa-miR-21-5p
	DNM1L	hsa-miR-21-5p
	FGF13	hsa-miR-486-5p
	GFPT1	hsa-miR-122-5p
	IGF1	hsa-miR-486-5p
	KLF6	hsa-miR-21-5p
	MAPK1	hsa-miR-122-5p
	NOD2	hsa-miR-122-5p
	NSA2	hsa-miR-21-5p
	PIK3R1	hsa-miR-486-5p
	PPARGC1A	hsa-miR-21-5p
	PPP1R8	hsa-miR-21-5p
	PTGS1	hsa-miR-21-5p
	RAPGEF5	hsa-miR-21-5p
	SEMA3A	hsa-miR-486-5p
	SMURF2	hsa-miR-486-5p
	SPARC	hsa-miR-21-5p
	STAT5A	hsa-miR-21-5p
	THBS1	hsa-miR-21-5p
	UCP3	hsa-miR-122-5p

**Table 7:** Protein-protein interactions of the selected gene list

Node1	Node2	Combined score	Confidence
CREB1	PPARGC1A	0.996	Highest
CREB1	MAPK1	0.962	Highest
PPARGC1A	UCP3	0.917	Highest
MAPK1	STAT5A	0.878	High
SPARC	THBS1	0.847	High
IGF1	PPARGC1A	0.764	High
FGF13	IGF1	0.744	High
DNM1L	PPARGC1A	0.734	High
CREB1	STAT5A	0.696	Medium
IGF1	STAT5A	0.666	Medium
MAPK1	PIK3R1	0.659	Medium
IGF1	THBS1	0.658	Medium
DNM1L	MAPK1	0.652	Medium
IGF1	MAPK1	0.621	Medium
CREB1	IGF1	0.593	Medium
FGF13	MAPK1	0.588	Medium
FGF13	THBS1	0.584	Medium
PIK3R1	STAT5A	0.58	Medium
CREB1	PIK3R1	0.558	Medium
IGF1	PIK3R1	0.524	Medium
IGF1	SPARC	0.478	Medium

## Data Availability

Data is available upon reasonable request from corresponding author.

## Abbreviations

ADA: American Diabetes Association
AMPK: 5’-activated protein kinase
CKD: Chronic kidney disease
CRP: C-reactive protein
CT: Cycle threshold
DKD: Diabetic kidney disease
EDTA: Ethylenediaminetetraacetic acid
ELISA: Immunosorbent assay
IL-6: Interleukin-6
IR: Insulin resistance
IRB: Institutional Research Board
NFAT5: Nuclear factor of activated T cells-5
OR: Odds ratio
PCR: The polymerase chain reaction
PTEN: Phosphatase and TENsin homolog
qRT-PCR: Real-Time Quantitative Reverse Transcription PCR
RQ: Relative quantification
SDS: Sequence Detection System
SMAD7: SMAD family member 7
T2DM: Type 2 diabetes mellitus
TLC: Total leukocyte count
TNF-α: Tumor necrosis factor-alpha
